# Exploring the link between viruses and cancer in companion animals: a comprehensive and comparative analysis

**DOI:** 10.1186/s13027-023-00518-7

**Published:** 2023-06-29

**Authors:** Francesca Parisi, Niccolò Fonti, Francesca Millanta, Giulia Freer, Mauro Pistello, Alessandro Poli

**Affiliations:** 1 Dipartimento di Scienze Veterinarie, Università di Pisa, Viale delle Piagge, 2, 56124 Pisa, Italy; 2 Dipartimento di Ricerca Traslazionale e delle Nuove Tecnologie in Medicina e Chirurgia, Università di Pisa, Via Risorgimento, 36, 56126 Pisa, Italy

**Keywords:** Cancer, Cat, Dog, FeLV, FIV, Gammaherpesvirus, Hepadnavirus, MMTV, Oncogenic viruses, Oncogenic mechanisms, Papillomavirus, Veterinary oncology

## Abstract

Currently, it is estimated that 15% of human neoplasms globally are caused by infectious agents, with new evidence emerging continuously. Multiple agents have been implicated in various forms of neoplasia, with viruses as the most frequent. In recent years, investigation on viral mechanisms underlying tumoral transformation in cancer development and progression are in the spotlight, both in human and veterinary oncology. Oncogenic viruses in veterinary medicine are of primary importance not only as original pathogens of pets, but also in the view of pets as models of human malignancies. Hence, this work will provide an overview of the main oncogenic viruses of companion animals, with brief notes of comparative medicine.

## Introduction

**Fig. 1:**
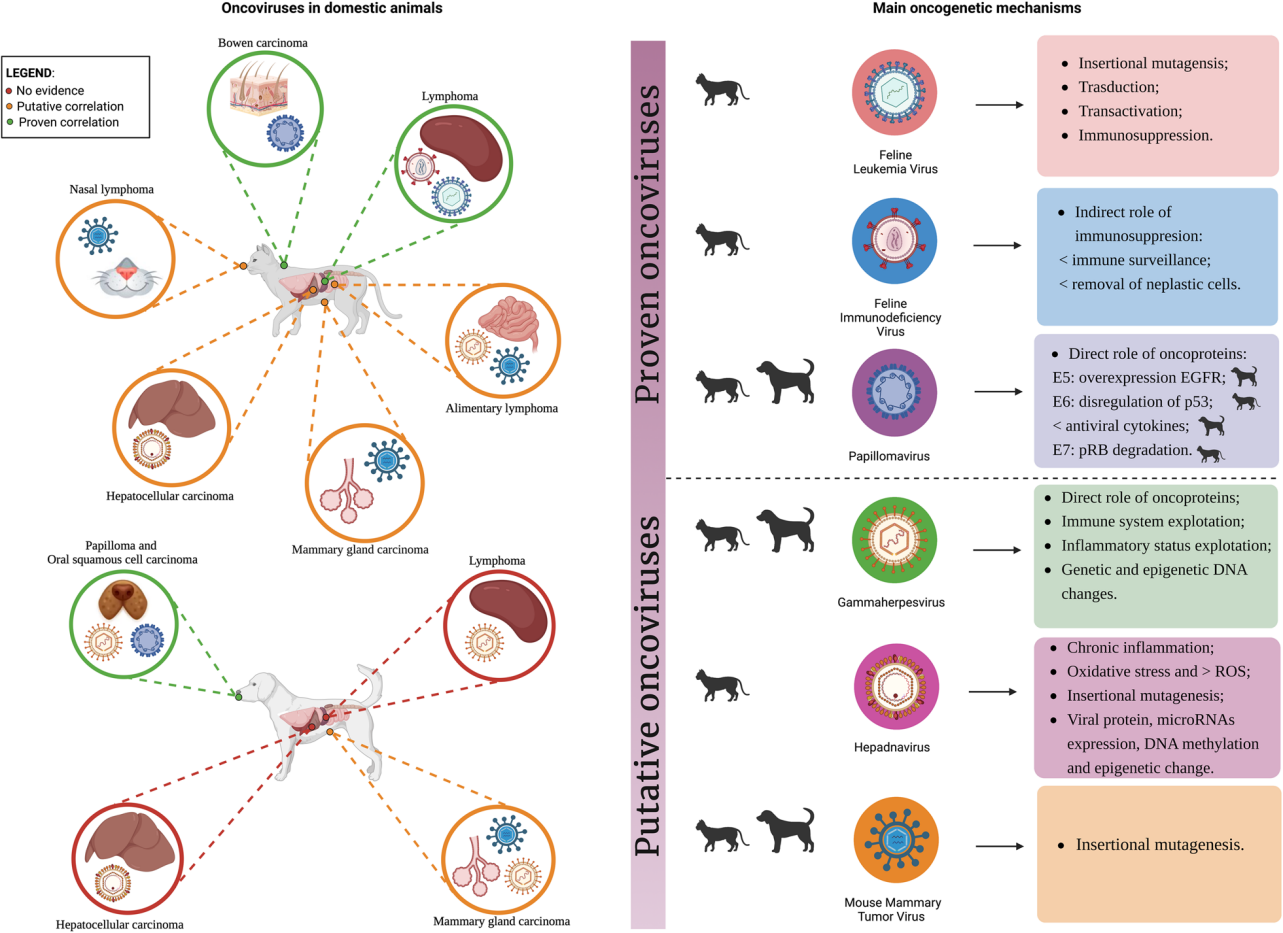
Overview of the oncoviruses in domestic animals and of their main oncogenic mechanisms. Created by Biorender.com

For many years, studies and observations on the animal world have contributed to our medical and scientific knowledge. For instance, porcine dissections were used to write the earliest anatomy textbooks around 900 A.D. [1], while rodents have been employed as models for cancer research for years [2]. Over time, animal models have yielded many general concepts in molecular oncology and hypotheses on the role of oncoviruses in tumor development [3]. Rous sarcoma virus, discovered by Rous in the early twentieth century, was the first oncogenic virus [4, 5] and a landmark in the oncovirus field. It led to the research on proto-oncogenes and the cancer-related action of tyrosine kinase. Nearly half a century later, in 1964, Epstein discovered the first human oncogenic virus, thanks to Rous's intuition [6]. Subsequently, it was discovered that oncoviruses are prevalent among both animals and humans and cause about 12% of human cancers [7–10]. Despite many similarities between animals and humans, there remain intrinsic differences that must be considered when they are used in cancer research [3]. However, the use of animals as models for human diseases is a crucial cornerstone of cancer research, both in the past and present, especially for prevention and therapy [3]. Over the years the use of animal models has been essential for medical understanding and advancement. To make only some examples, animals were extremely useful in the understanding process of human diseases, in the development of vaccines [11], antibiotics [12] and new surgical techniques [13, 14], in the identification and validation of new markers as well as in preclinical studies for the evaluation of new therapies [15]. The identification of a viral etiology for many types of cancers has several implications. It provides additional information on cancer development and progression and leads to the identification of new cellular targets for therapy. This knowledge is useful not only for virus-related cancers but also for those with no viral etiology. For example, the role of tumor suppressors p53 and pRb led to the development of several therapeutic approaches. Additionally, viral gene products themselves may serve as potential targets for therapy or may allow the development of screening and prevention strategies, as already happened with the Pap smear and vaccination against Human Papillomavirus (HPV)-related cervical cancer [16, 17]. Establishing a causal association between a virus and a specific cancer requires fulfilling most, if not all, of Hill's criteria [18], which demands a substantial number of epidemiological studies. For this reason, a limited number of viruses are currently considered as pathogens with oncogenic potential in humans. Among these, Epstein Barr Virus, Hepatitis B Virus (HBV); Hepatitis C Virus (HCV), Human Papilloma Virus (HPV), Human T-Lymphotropic Virus 1 (HTLV-1), Human Herpesvirus 8 (HHV-8), Merkel Cell Polyomavirus (MCP) and Mouse Mammary Tumor Virus (MMTV) are recognized [19, 20]. Furthermore, although there is a substantial body of literature on the correlation between viruses and cancer in domestic animals, it is often fragmented and lacks a comprehensive overview. Therefore, we consider it extremely useful to collect all data and review the various research studies carried out so far over the years. Modern oncology has focused on using companion animals as the best animal model for human diseases. Indeed, compared to rodents, they are phylogenetically closer to humans and show (i) similar genomic organization, (ii) similar cancer incidence, (iii) similar molecular pathological and clinical features, (iv) similar toxicity and therapeutic responses compared to the human counterpart. Of note, unlike laboratory rodents, they develop spontaneous cancer, and different from humans, they have a shorter life, making cancer progression often faster. Finally, they also share the environment and the socioeconomic factors with their owners [15]. All these features make the companion animals eligible to be the best candidates for animal models in cancer research, and for this reason we decided to focus this review on them. We will provide an overview of oncoviruses of veterinary importance in domestic animals, investigating their role as pathogens for both dogs and cats. In the first section of this review, we will describe the oncoviruses for which the oncogenic potential has already been proven in literature: Feline Leukemia Virus (FeLV), Feline Immunodeficiency Virus (FIV) and Papillomavirus (PV). In the second section, we will collect and discuss data about emerging potentially oncogenic viruses, for which a cause-effect relationship to cancer in domestic animals has not been established yet: Herpesvirus, Hepadnavirus, and Mouse Mammary Tumor Virus (MMTV). For all these viruses, the interest has arisen from evidence on the oncogenic potential of similar viruses in humans. For each virus discussed, we will provide data on their structure, life cycle, clinical presentation, transmission and oncogenic mechanisms in dogs and cats, when available, or in their original host when not, with brief comparative notes. An overview of the current knowledge about the association between viral agents and cancer in veterinary medicine is summarized in Fig. 1, together with a focus on the main oncogenic mechanisms proposed.

## Feline leukemia virus

FeLV is an exogenous retrovirus of the genus *Gammaretrovirus*. Infection occurs worldwide, with a prevalence different in various geographical areas, ranging from less than 1% to 20% based on cat density [21]. FeLV is an enveloped single stranded RNA virus made up of three genes, the group specific antigen (*gag*), the polymerase (*pol*) and the envelope (*env*) genes. These three genes are flanked by long terminal repeats (*LTR*s) containing promoter and enhancer elements. On the basis of the *env* sequences, three main subtypes are described which differ in receptors and cell tropism: FeLV-A, FeLV-B, and FeLV-C. Only the predominant subtype, FeLV-A, can be transmitted from cat to cat, while the other two result from genomic alterations achieved through different mechanisms, but they are typically not further transmissible. FeLV-B occurs in 50% of infected cats and it is the result of a genome recombination between the FeLV-A subtype and the endogenous FeLV-related retroviruses. Endogenous retroviruses in cats are common genetic elements resulting from retroviral infection of ancestors. These elements cannot be transmitted exogenously but are inherited vertically by the germ line [22]. The infection with FeLV-B subtype can accelerate the development of lymphoma or increase the virus neuropathogenicity [22]. FeLV-C subtype arises rarely (1%) after a point mutation in the *env* gene sequence and causes fatal anemia in infected subjects [22, 23]. A more recent discovery involved another FeLV variant that arises through multiple mutations of the env gene in FeLV-A-infected subjects. This variant, with a selective tropism for T-lymphocytes, is known as FeLV-T and is associated with severe immunodeficiency [24–26].

The receptor for the FeLV-A subtype was recently recognized as a thiamine transport protein [27], while the FeLV-B subtype enters cells using Na-dependent inorganic phosphatase transporters [26, 28], the FeLV-C subtype uses a hemopoietic cell-associated transporter molecule [29, 30], and the FeLV-T subtype uses a coreceptor expressed on T-lymphocytes called feLIX [31].

The mechanism of infection by FeLV is similar to those of other retroviruses. Once the viral glycoprotein spikes on the envelope surface recognize their specific receptors on cells, the viral core can be internalized. The two copies of single-stranded RNA released in the host cell cytoplasm are reverse transcribed into DNA by reverse transcriptase. The viral DNA enters the nucleus and integrates into the host cell DNA, acting as a template for the production of new viral particles [32].

FeLV is mainly transmitted horizontally through the oronasal route of infection [33] by contact with virus-spreading secretions (e.g. saliva) from viremic subjects [34, 35]. This route of transmission makes overcrowded environments, such as cat colonies and catteries, the most infectious places for cats, due to the sharing of food, water, bowls and mutual grooming [31]. After contacts with viremic cats, FeLV initially replicates in lymphocytes and macrophages in local lymphoid tissue of the oropharynx [21, 31]. Viral particles spread through draining lymph node and blood to tissues rich in rapidly dividing cells [36]. The outcome of infection may be variable, depending on a number of factors, like immune status and age of the host, concentration and pathogenicity of the virus, route of exposure, and the presence of concomitant diseases [21, 31, 37]. If the immune system is able to contain viral spread through an appropriate immune response, cats may recover from infection, being known as “regressor subjects” [21, 32, 38]. This event, in natural circumstances, takes place in 1% up to 10% of exposed cats [23]. In the remaining subjects, the immune response does not succeed in controlling virus replication and infected cats develop persistent viremia. Viral particles spread to the target organs (spleen, thymus, lymph nodes, and salivary glands) and cats manifest nonspecific clinical signs, like pyrexia and lethargy, becoming infectious to others [21, 37]. Generally, this phase is transient, lasting about three weeks, during which cats are known as “transiently viremic” [21, 37]. Most of these cats succeed in overcoming infection and develop an immune response protecting them from further new exposure. In those who do not succeed in neutralizing infection, the involvement of bone marrow has been reported. The virus integrates its genome in haemopoietic precursor cells, and the progeny cells released will all be infected, with the development of high-level viremia [37]. A number of subjects recover from viremia, but remain “latently infected” because of the persisting integration of the virus in bone marrow stem cell genome, while those who do not recover remain “persistently infected” [32].

Subjects with persistent infection (but also latently infected ones after reactivation [39, 40] may develop FeLV-related diseases and have a poor prognosis [23, 31]. Only 20% to 50% of cats with progressive infection were reported to survive three years post diagnosis [23, 41, 42]

FeLV-associated diseases are not only lymphoma and leukemia, as suggested by the name of the virus, but they also include non-neoplastic diseases related to its pancytotropism and to the involvement of bone marrow, lymphoid system and rapidly dividing cells. Among these diseases, myeloproliferative and immune-mediated diseases, cytopenia (particularly anemia), enteritis, and reproductive disorders can be found, along with secondary infections due to immunosuppression [23, 31, 32, 43, 44]

Lymphoma is the most common lymphoid malignancy associated to progressive infection by FeLV [45] and it is reported to occur in 10–20% of progressive infections [46–48]. FeLV-associated lymphomas are mainly high grade, T-type lymphomas, while their anatomic localization is related to the age of the host. The thymus is the most frequently involved especially in young cats [23], while FeLV-induced alimentary lymphomas are also frequent, but generally related to older subjects [31]. Spinal and multicentric localization are the most frequently reported sites [23], with the latter being typical of young subjects [31].

FeLV is a simple retrovirus, therefore its genome does not carry oncogenes directly causing malignancy, but it is suspected to cause cancer through indirect mechanisms. One of the most frequent ways is by insertional mutagenesis, through which the virus influences the expression of genes by integrating its genome in specific sites of the host DNA [23]. Particularly, integration may occur near cellular proto-oncogenes, which can be upregulated by enhancer sequences and transcriptional promoters located in the U3 region of LTRs [49–56]. Molecular analyses demonstrated that the most common integration sites (CISs) in FeLV-associated lymphomas occur in the vicinity of six genes, namely *c-myc, flvi-1, flvi-2, fit-1, pim-1, and flit-1* [53, 54, 57–61]. As evidence, mutational events in LTRs enhancing their functions [23, 62–65], as well as *c-myc* dysregulation [54], are frequently observed in FeLV-associated neoplasms. However, viral integration can also occur within sequences encoding tumor suppressing genes, interrupting their expression. In this case, both alleles should be inactivated to cause an effective gene loss of function, so this is an uncommon way [23].

Another mechanism responsible of the development of FeLV-related lymphomatous malignancies is transduction, thanks to which the virus acquires cellular oncogenes during its replication. In this eventuality, the oncogene will be transduced along with the viral genome [23, 61]. This mechanism is exploited by the FeLV-A subtype when it acquires cellular oncogenes like *fes, fms, fgr, abi,* and *kit* through transduction, giving rise to the so-called feline sarcoma viruses (FeSV), responsible of multicentric sarcomas [32, 66, 67]. FeSV belongs to the group of sarcoma viruses, recombinant viruses coming from the assembling of some leukemia virus genes and some host cell genes that Hardy, in 1981, defined as *sarc* [66]. Thus, FeSV genome is characterized by two distinct sequences subsets, one including sequences shared with FeLV helper virus and designated as *com*, the other including sequences coming from the transduction of host cell gene, known as *src* sequences*,* conferring transforming properties to the virus [68]. It has been highlighted that *src* sequences from sarcoma virus of the various species are close each other, thus suggesting that they came from the same ancient cellular genes that has been conserved for years in many species [66]. Furthermore, FeSV lacks *pol* gene and most of the *env* gene, thus it cannot synthetize reverse transcriptase enzyme and the proteins required for envelope production, like gp70 and p15E. For this reason, in nature FeSV exists as pseudotype virus with a FeLV helper [69–71]. Viral particles are composed by a FeSV RNA genome enclosed in a FeLV envelope. Thanks to the presence of envelope, viral particles can enter host cells through their receptors, within the cells the FeLV help reverse transcriptase allows the formation of both FeLV and FeSV proviral DNA, which are then integrated into the host cell DNA. In this way, viral genes may be expressed and the presence of *src* genes may allow malignant transformation of host cells, thus giving rise to sarcoma. Two distinct types of viral particles are then produced from these infected cells: FeLV viral particles, characterized by FeLV RNA and enclosed in FeLV envelope, and FeSV viral particles [66].

As last oncogenetic mechanism proposed, it has been hypothesized that FeLV may influence cellular genes through transactivation [72], by which transcripts from the U3 region may activate a signaling pathway commonly involved in oncogenesis. Moreover, the role of endogenous FeLV has recently been questioned, since two studies suggested that their presence was associated with better prognosis upon infection with exogenous FeLV [32]. Finally, we should also take into consideration that FeLV may contribute to the development of neoplasms simply by bringing about an immunosuppression state associated with viral infection which leads to the reduction of the immune surveillance and contributes to tumor cell survival [23].

## Feline immunodeficiency virus

FIV is an enveloped RNA virus belonging to the *Lentivirus* genus of the *Retroviridae* family [73]. It was described for the first time in 1986 in a cattery in California [74] and is known to cause an immunodeficiency syndrome similar to human AIDS in cats worldwide [75], with a prevalence ranging from 1% in healthy domestic cats up to 47% in feral subjects, with a higher prevalence in sick cats [32, 76–82].

Like other retroviruses, the FIV provirus is bordered by LTRs and comprises *gag, pol* and *env* genes, as well as other regulatory and accessory genes [83]. Based on the *env* gene sequence, six different subtypes of FIV are recognized, from A to F, with A, B and probably C as the most widely distributed subtypes. Recombinant subtypes have also been documented and the existence of further subtypes has been suspected [73].

To enter the host cells, FIV uses CD134 as its primary receptor and CXCR4, a chemokine receptor, as a secondary one. CD134 is expressed in CD4 + T-lymphocytes, B-lymphocytes and activated macrophages. For this reason, they act as the first target cells for the virus [73]. The mechanism of infection is similar to FeLV and other retroviruses (see the previous section). Proviral DNA may be transcriptionally active or silent, based on cellular environment [73]. FIV is transmitted through biting by infected cats [84]. This transmission route makes free ranging intact male cats, prone to fighting behaviors, more exposed to infection [31, 74]. Differently from natural conditions, vertical transmission has also been demonstrated in the experimental field [31, 32, 85, 86]. Unlike Human Immunodeficiency Virus (HIV), venereal transmission has not been documented for FIV, but viral particles have been found in semen and experimental infection through the vagina has been demonstrated. Moreover, transmission through viral inoculation in bloodstream is thought to be an additional route [32, 73].

As HIV, FIV can progress through several stages of infection. Viral particles are inoculated through saliva, infect lymphoid and myelomonocytic cells, particularly CD4 + T lymphocytes, macrophages, and dendritic cells, integrate their genome, and then start to replicate and release new virions. In the first phase of viremia peak, early clinical signs are fever, anorexia, depression, leucopenia, gingivitis, and general lymphadenopathy [31, 75], which regress with the development of the host immune response. Within one week from infection, FIV-specific CD8 + T lymphocytes can be detected [87], while anti-FIV antibodies and virus-neutralizing antibodies are detected by three weeks after [88]. The development of a specific immune response causes the viral load to decrease until a steady state is reached, which marks the beginning of the latent asymptomatic phase. This stage is characterized by a slow and steady reduction of CD4 + T lymphocytes that may last for years, in some cases for the entire life of cats, while the infected animals are infected subclinical [31, 75]. However, an evolution to an end stage with clinical manifestation similar to AIDS for HIV-infected humans is also possible, with marked loss of CD4 + T lymphocytes, frequent bacterial respiratory, ocular and oral infections, enteritis, neurologic disorders, opportunistic infections, and development of cancers [31, 75]. Despite this possible evolution, infection is not necessarily life-threatening, and many studies demonstrated that life expectancy of FIV-infected cats is quite comparable to that of non-infected ones [42, 89, 90]. Even if the outcome of FIV infection is not predictable [90–92], it has been suggested that several factors influence its evolution, like genetic features and concurrent diseases [31]. The development of cancer is reported with an incidence from 1 to 21% in positive subjects. In addition to sporadic reports of myeloproliferative leukemia, mastocytoma, fibrosarcoma, and squamous cell carcinoma [93–95], lymphoma is the most common neoplasia associated with FIV [76, 84, 94–101]. Particularly, it has been reported that FIV-infected cats are 5 to 6 times (up to 80 times in case of FeLV/FIV coinfection) more susceptible to develop lymphoma compared to uninfected ones [48]. FIV-associated lymphomas are reported to be mainly B-type [100–105] with a prevalence from 40 to 87% [102–105], while T-cell lymphomas range between 0 and 28% [101, 103–105] and non-B, non-T lymphomas are uncommon [101, 103–105]. A possible explanation of the prevalence of B-type lymphomas is the rapid proliferation of B lymphocytes occurring in the early phase of infection [75], that statistically increases the chances of these cells to malignant transformation [106–108]. There is no anatomical predisposition, since FIV-associated lymphomas have been described in a multitude of organs [75], and neoplasms arise in cats ranging from 5 to 13 years [48, 93, 94, 101–103, 109]. In respect to oncogenic mechanisms, FIV is not likely to be directly involved in lymphomagenesis because its genome does not carry oncogenes, so it cannot induce neoplasms through acute transformation. Insertional mutagenesis mechanisms are also unlikely since, in this perspective, clonal integration of provirus should be recognized in lymphomas. Only two studies support this finding [105, 110, 111], while most of the other investigations trying to confirm insertional mutagenesis power of FIV have failed in their attempt [99, 101, 103]. Nowadays, the most accepted theory is that FIV induces lymphoma via indirect mechanisms, for instance through the reduction of immune surveillance, and consequent impairment of neoplastic cell removal secondary to virally-induced immune dysfunction [75].

## Papillomavirus

PV has become relevant in human medicine since it was proved that HPV type 16 and 18 are causative agents of cancer. Particularly, it has been shown that some genotypes, belonging to *Alphapapillomavirus*, are able to induce cervical cancer, as well as anogenital tumors and a percentage of head and neck squamous cell carcinomas mostly in the oropharyngeal area [112–115]. Following these findings, nowadays, human PVs (HPVs) are subdivided in “low-risk types”, causing asymptomatic or self-resolving conditions, and “high-risk types”, causing cancer [116]. There is more and more compelling evidence that canine papillomavirus (CPV) and feline papillomavirus (FPV) may also cause cancer in dogs and cats, respectively, but additional studies are required to investigate their role in tumor development [117]. Although viral etiology of warts has been recognized since 1907 [118], the first association between PV and cancer dates back to 1935, after one study on rabbits [119]. It was only in 1981 that PVs acquired great importance, when zur Hausen et al. proved that PV caused cervical cancer in humans [120]. PV was suspected as a viral etiological agent for cancer for the first time in 1969 for dogs [117] and in 1990 for cats [121], but it was only in 1994 that the first canine PV was sequenced [122], while the first feline PV was sequenced later, in 2002 [123, 124].

PV is a small, non-enveloped virus containing a double-stranded circular DNA genome, composed of three main regions: the long control region (LCR), the early region (ER) and the late region (LR). The LCR is involved in the modulation of viral replication and transcription. The ER and LR are composed of six and two open reading frames (ORF) respectively, in detail E1, E2, E4, E5, E6, E7 for ER and L1 and L2 for LR, encoding for their respective and homonymous proteins [125, 126]. Particularly, E5, E6 and E7 are oncoproteins responsible for the induction of cell proliferation and cancer development through different mechanisms. Differently from HPV and CPV, FPV doesn’t express E4 and E5 proteins [127]. Taxonomic classification of PVs is based on the highly conserved L1 ORF. If the similarity between L1 ORF is more than 60% then two PVs are classified within the same genus; if similarity is less than 90%, they are classified in different types [117, 128]. Types belonging to the same genus generally infect the same host species with similar presentation [128]. In dogs, twenty-three types of CPV, belonging to the *Lambdapapillomavirus*, *Taupapillomavirus* and *Chipapillomavirus* genera, are reported, while, in cats, six types of FPV have been detected within the *Lambdapapillomavirus*, *Taupapillomavirus* and *Dyothetapapillomavirus* genera [116].

PVs can be transmitted directly, through contact with infected subjects, or indirectly, via fomites [116, 117]. The infection begins when abrasions on mucocutaneous epithelium allow the virus to access the basal layer of cells. Here, binding between L1 protein and heparan sulfate proteoglycan receptors allows conformational changes in viral capsid, thus permitting contact between the L2 viral protein and a second receptor, located in the annexin A2 heterotetramer [129, 130]. PV internalization occurs through endocytosis: the L1-L2-viral DNA complex is transported at first to the Golgi network and then to the host cell nucleus [131]. Here, viral replication and transcription occur, thanks to cellular transcription factors (TFs), which are specific of differentiated epidermal cells [132]. Thus, as basal cells differentiate, viral genome is amplified, and early proteins are expressed. Particularly, E6 and E7 oncoproteins increase their expression and interact with p53 and pRB, with mechanisms explained in detail below, inhibiting apoptosis, causing cell cycle arrest and enhancing the progression from G1 to S phase in cell cycle [133, 134]. In the meantime, E1 and E2 expression amplifies viral genome replication and multiple viral DNA copies are produced [135]. In this way, PV infection persists since progeny cells inherit PV DNA and move to the suprabasal layer of the epithelium [116, 136, 137]. The production of infectious viral particles is possible only once L1 and L2 are expressed and produce the viral capsid around viral DNA. Then virions can be released into the environment thanks to the action of protein E4, which is responsible of viral particle-laden keratinocyte rupture [138–140].

Clinical presentation of PV infection may be variable. Generally, most HPV types do not cause clinical manifestations [141–143]; infection is subclinical for most dogs and cats as well [144–146]. Only a small percentage of infection cases exhibits an increase in keratinocytes replication hesitating in epithelial hyperplasia and papillomas [117]. This outcome is generally linked to the presence of certain PV types or to the lack of immune response. Indeed, even if the virus is confined in the outer epithelial layers and the immune response is weak, PV elicits both a humoral and cell-mediated response. The former is responsible of the production of IgG antibodies blocking further infection by the same PV-type, the latter is involved in resolution of an established infection [117]. Conversely, immunosuppression may favor the progression of PV infection into clinical disease, with different presentations: hyperplastic lesions, preneoplastic diseases and cancers.

Among hyperplastic lesions, PV is frequently associated with cutaneous and oromucosal warts. This clinical presentation is typical of young dogs [147], where CPV2 and CPV1, alone or in coinfection, and rarely CPV6, cause papillomas [148–152]. They usually arise as cauliflower-like lesions on areas subjected to trauma, like feet or around the face, lips, and ears [116, 117, 153–155] and are generally self-limiting. There are very few reports of CPV-induced warts transformed into SCC [156]. However, there are some reports of lesions that continued to increase in size, spreading to the haired skin [157] or progressing to SCC [158].

Surgical excision or cryotherapy is recommended if these lesions become too large and interfere with eating or breathing [159]. In cats, however, this clinical presentation is not so frequent: warts are suspected to be caused by FPV1. There are very few reports on this manifestation appearing on the nasal planum, on the eyelid or arising in clusters on the ventral side of tongue [116, 160, 161].

PV-related preneoplastic lesions in domestic animals are represented by pigmented viral plaques both in dogs and cats and by Bowenoid in situ carcinomas (BISCs) in cats alone. Of note, pigmented viral plaques and BISCs are generally described as different pathologies even if they probably represent different stages of the same entity. Particularly, it is the presence of more marked hyperplasia that makes the lesion bulge into the dermis, keratinocytes dysplasia or basal cell crowding help to identify BISCs [162].

PV-related preneoplastic lesions rarely develop both in middle-aged and older dogs and cats [163, 164], and are caused by Chipapillomavirus types, mainly CPV4 in dogs [165–168], and FPV2 or, less frequently, other Taupapillomavirus in cats [162, 169–173]. In both species, infection is generally subclinical, but immune disfunctions may cause inability to limit viral replication, leading to increased PV replication and thickening of the epidermis, with the development of lesions. In both species, breed predisposition has been proposed as linked to inherited deficiency in keratinocyte immunity. Particularly, predisposition has been highlighted in Vizla and Pug dogs [164, 165, 174, 175] and in Sphinx and Devon Rex cats [176, 177]. Plaques usually occur on limbs and face in dogs, while preneoplastic lesions generally arise on the head or neck in cats. Canine plaques have a benign course, where coalescence or extension to other areas are rare [163, 178] and spontaneous regression often occurs. Progression to SCC has been reported only once, during infection with specific HPV types, like CPV16 [149, 151, 179–181]. Conversely, in cats, these plaques rarely regress; quite oppositely, they usually progress to ulceration and more severe morbidity that has to be treated through cryotherapy, surgery or using specific cremes [182]. Progression to SCC has been reported, especially in Sphynx and Devon Rex cats [176, 177].

Due to the frequent subclinical course of CPV, even if some studies report the presence of CPV DNA in canine SCCs [183–185], its detection is challenging to understand. Conversely, several studies have linked FPV to SCC, basal cell carcinoma and Merkel cell carcinoma in cats [117, 186]. Particularly, literature suggested that an FPV etiology may be suspected in 75% of feline SCCs arisen in UV-protected areas, while in UV-exposed areas SCCs are mainly caused by UV rays and only 30% of them are linked to PV [187]. The most frequent SCC-associated type in cats is FPV2 [188–193] which is reported to cause tumors. A key role is played by viral E6 and E7 proteins, that dysregulate normal p53 function and degrade pRb, leading to impaired recognition of damaged DNA and disruption of important cell replication checkpoints [190, 194]. Despite FPV2 infects cats from the birth and lifelong, not all infected subjects develop SCC [195]. This evidence suggests that other factors may be involved in the development of this neoplasm. Moreover, it is not clear yet if PV-related cancer evolves necessarily from PV-induced plaques, or if it may arise on skin without precursor lesions [164, 176, 177]. Among the diseases recognized to be caused by PV there are also feline sarcoids. Like equine sarcoids, they are caused by aberrant infections with Bos taurus papillomavirus (BPV) [196, 197]. The etiological agent causing feline sarcoids is a delta papillomavirus previously known as FeSarPV. Nowadays it has been classified as BPV-14 and it has been highlighted is closer relation with BVP types 1, 2 and 13 [196, 197]. Feline sarcoids are mesenchymal neoplasms characterized by fibroblasts proliferation with epithelial hyperplasia and deep rete ridges [198]. They generally arise on face or digit, probably because these sites are frequently predisposed to trauma which allow viral penetration in the dermis [198]. Feline sarcoids are very rarely reported. One possible reason for these limited reports can be linked to misdiagnosis, since if only one portion of the tumor is submitted to histopathological analysis, without including epithelium, it is difficult to diagnose correctly. Moreover, as already discussed, this is a typical finding of cats living in country environment, which are often feral or subjects receiving less care than the housed ones [197]. Finally, sarcoids can spontaneously evolve in regressive lesions, further reducing the frequency of this finding [197].

The oncogenic mechanisms of PV are well known in humans, and there is strong evidence of similar pathways in dogs and cats. The integration of viral DNA is a preliminary and essential step in the oncogenetic process [199], as previously suggested by studies that linked it to both viral and host genome instability caused by oncoproteins E6 and E7, leading to double strand breaks [200]. For this reason, even though all viral genome is essential to the virus’s life cycle and regulation, oncoproteins E5, E6 and E7 have a key role in the process of cancer development [201].

It has been reported that E6 interacts with two different classes of molecules: proteins harboring the LxxLL motif and proteins with a PDZ domain. The ligase E6-associated protein belongs to the former class and, when associated to protein E6, is involved in p53 degradation through the proteasome, thus inducing cancer. This kind of interaction occurs both in low- and high-risk types. On the other hand, proteins containing PDZ domains are involved in many cell signaling pathways and their association to protein E6 is closely linked to malignant cell transformation [202]. Interaction of E6 with PDZ proteins is a prerogative of high-risk PV types, and, alone, it is sufficient to cause neoplasms through the induction of cell transformation [140]. These two mechanisms are common in human, canine and feline species, even though the ability of E6 to dysregulate p53 should be further investigated in dogs [140]. Furthermore, protein E6 has also been described to affect the production of antiviral cytokines in keratinocytes of immunodeficient dogs. In humans, E6 has also been reported to decrease Bax and Bak protein family content, preventing them from entering mitochondria and avoiding the activation of apoptosis [165].

In humans, E7 protein acts as oncogenic through proteasome-mediated pRB degradation [203]. pRB is a tumor suppressing protein whose degradation releases E2F and promotes the entry of the infected cells in the S phase of cell cycle. In cats, E7 acts in a similar way, while in dogs, E7 lacks the pRB binding site, and therefore, PV has been suggested to modify the cell cycle in other ways [140, 195]. E5 is the smallest and least studied PV oncoprotein and its oncogenic action is linked to its promoting activity on the expression of the epidermal growth factor receptor (EGFR). Moreover, in humans, E5 can bind endosomal vacuolar V-ATPase, affecting its activity and reducing vesicular transport [204]; it also promotes degradation of Bax, a proapoptotic protein, thus preventing apoptosis [205].

The case of BPV-14, etiological agent of feline sarcoids, deserves a separated discussion. Even if the oncogenic mechanism has not been fully understood yet, it has been recognized that E5 protein produced by delta BPVs has a key role in the neoplastic transformation process [196, 197]. This eventuality is linked to the binding and the activation of the platelet derived growth factor beta receptor (PDGFB-R), which is possible thanks to the presence of 4 specific amino acids within the E5 protein. This binding causes a cascade of events, like the activation of kinases and further mechanisms to support neoangiogenesis and immune evasion, leading to mesenchymal cell proliferation [206–208]. On the other hand, E6 protein lacks PDZ binding motif and E7 lacks retinoblastoma binding sites, further elements arguing in favor of an essential role of E5 protein in the BPV-14 caused oncogenic transformation [196].

Assuming that in recent years feline oral squamous cells carcinoma (FOSCC) are thought to be very similar to human head and neck squamous cells carcinoma (HNSCC) [15], and the role of FPV-2 as etiological factor for the development of these neoplasms is supported by several studies, new evidence suggests an emerging role of cats as spontaneous model for HPV related HNSCC [209]. The reasons for this speculation have their root in several pieces of evidence coming from a multitude of studies collected and revised by Altamura and Borzacchiello [210]. As already known, HPV-positive SCC are classified as distinct entities because they showed different molecular, genetic, and biological features than their HPV-negative counterpart. It is not yet clear whether the same is applicable to FOSCC. Given that this is one of the last pieces missing to the full confirmation of the reliability of such a model, Altamura and Borzacchiello launched an appeal for a collective effort in additional research and data collection on FPV [210]. Furthermore, a coordinated interest of researchers should also be promoted in the light to develop further preventing therapies, both for companion animals and humans. Indeed, it is noteworthy that prevention, one of the core principles of modern oncology, reached a milestone exactly in contrasting human papillomavirus, since a vaccination campaign has already been active for some years targeting adolescent girls. Vaccine effectiveness has been demonstrated to be high since the prevalence of HPV type 6/11/16/18 infection and, of consequence, the prevalence and incidence of genital warts, decreased in the targeting population [211], a great achievement that encourages the research effort on this area.

## Gammaherpesvirus

With this paragraph, we will begin the discussion of viruses for which an oncogenic potential has already been confirmed in other species while they are currently under investigation in cats and dogs. For this reason, in this and the next two sections, we will start from solid data already present in the literature in the original species, while attempting to retrace the studies carried out in dogs and cats so far. Herpesviruses are the first viral agents described as oncogenic in human medicine and classified among class I carcinogens by the International Agency for Research on Cancer (IARC) [212]. They are double-stranded DNA viruses classified into three different subfamilies, *Alfaherpesvirinae*, *Betaherpesvirinae* and *Gammaherpevirinae* [213]. In humans, two Gammaharpesvirus are frequently associated with HIV-related neoplasms [214, 215], namely Epstein-Barr virus (EBV, Human Herpesvirus 4) and Kaposi’s sarcoma-associated herpesvirus (KSHV, Human Herpesvirus 8). KSHV belongs to the *Rhadinovirus* genus, it is responsible for epidemic Kaposi’s sarcoma, frequently associated with HIV infection [216, 217]. The EBV belongs to the *Lymphocryptovirus* genus [218]: nowadays, its causal role in transforming latently infected lymphocytes in HIV-positive patients is well described [214]. Although the infection is generally asymptomatic in immune-competent individuals during childhood [219, 220], infectious mononucleosis may be a manifestation in adolescents and early adults [221]. Furthermore, this virus has been linked to the development of some lymphoproliferative neoplasms, for instance Burkitt’s lymphoma, Hodgkin’s disease, diffuse large B cell lymphoma, T-cell lymphoma, [222, 223] and nasopharyngeal and gastric carcinoma [224, 225], since lymphoid and epithelial cells are both highly receptive and permissive for infection [226, 227]. From 1995 to the present days, a great number of studies suggests that EBV may also have a role in the development of breast cancer (BC) in women [228–245] alone or in association with other viruses [246, 247]. However, other authors did not succeed in confirming this association [229, 242, 248–253] and, despite the fact that epidemiological evidence points out a higher risk of BC in the presence of EBV, its role in the development of BC remains controversial [254]. A search for the involvement of EBV or an EBV-like virus in BC, as well as in similar feline and canine types of tumors, is under investigation.

EBV is characterized by a 184-kbp genome encoding more than 85 genes, including numerous oncogenes such as nuclear antigens (EBNA1, -2, -3A, -3B, -3, C, -LP), latent membrane proteins ((LMP)-1, -2A, -2B), and noncoding RNAs (EBERs and miRNAs) [255]. During infection, EBV alternates between two possible states, i.e. latent and lytic state. In the latent state, the virus genome exists as a closed circular plasmid DNA in the cell nucleus, incorporated with histones, and replicates at the same time as the host genome. It is inherited by progeny cells, and only a select number of genes are expressed, depending on infected tissue, state of cells, and immune condition [256–259].

Latency factors play a key role in cancer development as they promote cell proliferation. Three patterns of latency have been reported in humans. Burkitt lymphoma and gastric carcinoma generally undergo type I latency, where gene expression is limited to EBERs and EBNA1. Some Hodgkin lymphomas, nasopharyngeal carcinomas (NPC), and T/NK lymphomas express the previous genes together with LMP1 and LMP2 genes in the so-called type II latency. EBNA2, EBNA3, EBNA-LP, together with all the previous type I and type II genes, are typical of post-transplant lymphoproliferative disorders and lymphoblastoid cell lines (LCLs) in type III latency.

In the lytic cycle, all the viral genes are expressed at the same time to produce new virions. It has been suggested that, at the beginning of infection, EBV follows an abortive cycle, during which only the immediate-early and early genes are expressed without DNA replication. This short cycle is then silenced after a few weeks, and a latent phase begins. A number of cells may go back from the latent stage to an abortive phase and then become silenced again, while others may express late genes and replicate the viral genome, thus entering the complete lytic cycle, with production of new virions [260].

However, the lytic stage is also involved in carcinogenesis, as it causes intense production of cytokines and growth factors that are exploited by neoplastic cells for their metabolism. In humans, EBV oncogenesis has been reported to occur through three different mechanisms. Firstly, the direct activity of viral oncogenes (such as LMP-1 and -2, EBV nuclear antigens (EBNAs), EBV-encoded nonconding RNAs (EBERs) and microRNAs) is implicated by eliciting growth-promoting signals or mitogen-activated protein kinase, or by silencing tumor suppressors, with several mechanisms which have not yet been fully understood [261–268]. Secondly, an indirect effect occurs through suppression of the host immune system (e.g., systemic immunosuppression during AIDS or associated with transplant, B cell lymphoma and disorders causing downregulation of MHC molecules) [217, 269, 270], or by exploitation of the inflammatory status, when cytokines and growth factors are released to promote neoplastic growth. Finally, EBV oncogenesis may also be linked to genetic or epigenetic alterations of host DNA, such as *myc* traslocation in Burkitt lymphoma [257, 272] or introducing mutations in suppressing genes leading to their inactivation [263, 267, 273]. For more comprehensive reviews on the topic see Murata et al., 2014 [260]

The close relationship between pets and humans suggests that an EBV-related infection might exist in domestic animals. In 2005, Chiou et al. found an 88% prevalence of anti-EBV antibodies in 36 pet dog blood samples and identified the presence of an EBV-specific BamHI W fragment in 71% of the corresponding 21 leukocyte DNA samples. In addition, through in situ hybridization (ISH), they also highlighted EBER in dog blood and bone marrow [274]. In 2010, Milman et al. detected anti-EBV antibodies in 43/112 in dog sera coming from UK, 67/104 in canine serum samples from US, EBV-like sequences in 1/33 canine palatine tonsil and succeeded in confirmed their presence in 38/100 cat blood samples, too [275]. However, EBV was detected molecularly in tissues from one dog, while RT-PCR did not succeed in detecting transcripts associated with lytic infection or latency, thus suggesting that pets are exposed to EBV or EBV-related viruses, but with no signs of persistent infection in the analyzed tissues. Interestingly, the prevalence of exposure is higher in pet cats rather than in stray ones, suggesting the owners as the main source of virus.

Later research focused on dogs with tumors. In 2012, Huang et al. reported the presence of anti-EBV antibody both in healthy subjects and in those with spontaneous lymphoma, together with the finding of extracellular viral particles similar to EBV in cultures of canine malignant B cells. This suggested that an EBV-like virus could be involved in some steps of canine lymphomagenesis [276]. A significant step forward was made in 2013 by Chiu et al., who restricted their study to dogs with tumors, particularly focusing on oral tumors, since it had already been proven that EBV is transmitted through saliva in humans [277–279]. They found 80% of EBV DNA prevalence in 10 samples from canine oral tumors, together with the presence of transcripts of EBER, of the viral bcl2 homologue BHRF1, and of LMP1. Moreover, they confirmed the presence of virions by transmission electron microscopy, highlighting a similarity in size between these virions and EBV viral particles [280, 281]. Furthermore, the similarity of the canine EBV-like virus LMP1 and the identity of canine BHRB1 both to the EBV counterparts argued in favor of a close relationship between canine and human viruses [279].

Despite all these encouraging results, another recent study by Waugh et al. [282] did not find an association between EBV or a related virus and 112 samples from canine lymphomas. However, through serological analysis, these authors confirmed that EBV or a closely related virus circulates in dogs, albeit with a lower prevalence than the one previously found. Although dogs with cancers were more likely to be seropositive than the others, the authors did not find higher antibody titers in subjects with lymphomas, compared with those bearing other types of neoplasms, nor did they find different seroprevalences between dogs with B- and T-cell type lymphomas, as expected. Starting from the idea that herpesviruses are extremely species-specific and rarely cross species barrier, they also set up multiple degenerated PCR assays to evaluate the presence of other herpesviruses, related but different from EBV, in canine samples. Again, they found no evidence of the involvement of a Gammaherpesvirus in common canine lymphoma. Recently, EBV was suggested as a putative etiological agent in the development of breast cancer [283]. For this reason, a recent study focused on the search for the putative involvement of an EBV-like Gammaherpesvirus in canine mammary tumors, once it was found to infect dogs (as mentioned above). However, only one sample was found to be positive for EBV genes. Although the authors suggested that their results could have been affected by technical factors related to DNA quantity or quality or other unclear factors, they had to conclude that there was not sufficient evidence of EBV involvement in the carcinogenesis of canine mammary tumors [284].

Due to the close parallel between HIV and FIV, and the frequent association between EBV and HIV infections, a similar causal role for a putative Gammaherpesvirus in cancer is under investigation in cats. A novel feline Gammaherpesvirus, the *Felis catus* Gammaherpesvirus 1 (FcaGHV-1,) has been recently discovered [285]. Subsequent studies have demonstrated a worldwide prevalence ranging from 5% up to 25% based on the geographical area considered [286, 287]. It has been hypothesized that this virus is transmitted horizontally, sharing its route of infection through biting with FIV [188], and that age, male sex and concurrent FeLV or FIV infection may be predisposing factors for cats [260–262]. Further studies are needed to confirm these observations.

In a study, FcaGHV-1 DNA was detected in 40.4% out of 104 FIV infected cats and it was estimated that a FIV-positive and haemoplasma-positive subjects was respectively 4, 5 [286, 287] and 16 times [288] more likely to be FcaGHV1 positive compared to the negative cats [288]. FcaGHV-1 viral particles have been also detected in the bone marrow and small intestine of cats with alimentary lymphoma [286]. In one of those cases, researchers succeeded in demonstrating and isolating the viral genome through quantitative PCR (qPCR) [213]. More recently, Aghazadeh et al. highlighted FcaGHV-1 DNA in one FIV-associated alimentary lymphoma through in situ hybridization (ISH) [289]. To sum up, even if the available data support a pathogenic role for this virus, at least in cats, further studies are needed to elucidate its involvement in oncogenesis.

## Hepadnavirus

The discovery of a Hepatitis B-like virus in domestic animals is relatively recent. Its prototype is the Hepatitis B virus (HBV), which has strict tropism for the liver and causes chronic hepatitis and hepatocellular carcinoma (HCC) in humans [290]. In 2018, a new virus was detected for the first time in an Australian FIV-positive cat during transcriptomics studies [291]. This virus has been named Domestic Cat Hepadnavirus (DCH) and was classified as a circular, partially double stranded DNA virus belonging to the *Orthohepadnavirus* genus, *Hepadnaviridae* family. Similar to the others Hepadnaviruses, its genome encodes four overlapping reading frames (ORFs) for the polymerase (P), surface (S), core (C), and X proteins [291, 292]. Although DCH belongs to the same genus as HBV, the real function of DCH viral proteins should be elucidated due to the phylogenetic divergence between DCH and HBV [291, 293, 294].

HBV oncogenesis is a multifactorial process. The first mechanism of HBV-related HCC induction is linked to chronic inflammation. HBV infection induces chronic inflammation in the liver, leading to regulatory T cell (Treg) disfunction, increased cytokine production (TGF-ß, IL-4, IL-10, IL-12, IL-13) and alteration of specific signaling pathways that ultimately increase the risk of hepatocarcinogenesis. Furthermore, HBV can cause oxidative stress with increased ROS production and ROS-induced DNA damage, frequently associated with chromosomal aberrations and cellular transformation. HBV-DNA integrations have been detected close to certain gene targets, suggesting that viral DNA integration in the host genome may be another way through which HBV can drive malignant transformation. Lastly, other specific mechanisms involving selected viral proteins (HBs and HBx), microRNA expression, DNA methylation modifications, and related epigenetic changes have been associated with HCC development. For a more comprehensive review on the issue, see Stella et al. [295].

Over the years, molecular studies have been carried out in several countries to test the prevalence of DCH in cat populations. Recent studies highlighted that 6.5% sera of 123 Australian cats [289] and 10.8% of 390 Italian ones [296] were DCH-positive and showed active viremia. In Malaysia, DCH has been detected in 12.3% of 253 blood samples and 14.9% of 87 liver samples [297]. In Thailand, DCH was confirmed in 12.4% out of 209 sera and in 20% of both sera and organs from 15 necropsies [294]. A multicentric study including 86 liver biopsies samples from the United States, United Kingdom, Australia, and New Zealand confirmed the presence of DCH DNA in cats with selected pathologies [298]. In 2022, a Japanese PCR-based study found a lower prevalence of 0.78% in 139 feline blood samples [299]. Furthermore, as HBV and Hepatitis C virus co-infection in humans was hypothesized to increase the risk of uveitis [300], DCH was also analyzed in subjects with uveitis where the virus was detected in 2 out of 65 cats, while the healthy control group resulted to be negative [301].

Given the possibility of inter-species transmission, the evidence that some viruses circulate among both dogs and cats [302], and the presence of antibodies specific to HBV in canine sera from several studies [303–306], it was hypothesized that HBV-like viruses might also be harbored by dogs. In 2019, Hepadnavirus DNA was detected in dogs for the first time using PCR targeting the preS/S1 genomic region and the core gene of HBV. Hepadnavirus DNA was identified in 10% of sampled dogs [306]. In 2022, another study reported that 6.3% of canine sera samples tested positive by qPCR, and the virus became known as Domestic Dog Hepadnavirus (DDH) [307]. DDH genome showed 98.0% nucleotide identity at the whole genome level with the Italian DCH and 96.9% with the Australian one [308].

Over time, similarities have been described between Hepadnaviruses infecting domestic animals and HBV. Piewbang et al. suggested that the localization and maturation of Dane-like particles in liver of cats analyzed by transmission electron microscopy was ultra-structurally similar to those observed for HBV [294, 309, 310]. Moreover, different studies involving infected cats have reported an elevation in liver disease-related markers (such as serum alanine aminotransferase) similar to HBV-infected humans [296, 297, 311] leading to the suspicion that DCH in cats may reflect similar tropism and pathogenesis to that of HBV in humans [294]. This suspicion prompted Pesavento et al. [298] to focus their research on a putative correlation between DCH and liver damage in cats. They analyzed liver biopsies from healthy (n = 15) and diseased (n = 71) cats through PCR and ISH and reported that a conspicuous percentage of subjects with liver diseases already reported to be associated to HBV infection in humans were positive for DCH, namely 43% (6/14) of chronic hepatitis cases and 28% (8/29) of HCCs. Conversely, subjects with bile duct-associated liver disease and healthy ones were negative for DCH.

Histologic features of hepatitis and hepatic neoplasia in cats have been described as extremely similar to those observed in HBV-related diseases in humans, strengthening the speculation that DCH may be associated with hepatitis and HCC in cats. Similar alterations in liver disease-related markers have been highlighted in DDH-positive dogs as well [307], but Choi et al. [312] did not succeed in confirming a contribution of Hepadnavirus in either chronic hepatitis or HCC [312]. Data from all these studies suggest that DCH infection is associated with immunocompromised conditions in cats [291, 293, 295, 296]. Moreover, due to viral particle detection in serum, whole blood, heart, lungs, intestines, kidneys, and spleen, [291, 293, 295, 296] researchers hypothesize that DCH may spread from one cat to another through blood, but not through semen or percutaneously, as for HVB infection [308, 313]. These observations were also supported by the longitudinal observation of two DCH-positive cats that constantly yielded negative DCH amplification PCR results of oral, conjunctival, and preputial swabs [314]. Regarding fecal transmission, although Capozza et al. [314] found only negative rectal swabs in their longitudinal studies, Piewbang et al. [294] found high numbers of DCH genome copies in intestinal samples and the expression of viral protein through IHC in cells from intestinal villi of cats. However, due to the limited numbers of studies, knowledge on the pathobiology of DCH and DDH is inconclusive, and whether these viruses are apathogenic or may have a potential role in certain clinical diseases remains to be elucidated.

## Mouse mammary tumor virus

MMTV infection is presently considered a proven risk factor for the development of mammary carcinoma in mice. Its discovery dates back to 1936, when John Bittner highlighted that mice with mammary carcinoma could transmit a factor capable of causing the development of the same cancer in the offspring that fed on infected milk [315]. This agent, known from the beginning as “milk factor”, was classified as a retrovirus in 1966 and later became known as Mouse Mammary Tumor Virus. Since its discovery, speculation has been made about the existence of a similar viral agent linked to human breast cancer (BC) first, and to neoplasms of domestic animals later. MMTV is a Betaretrovirus of the *Retroviridae* family. While it was initially classified as a simple retrovirus, now it is classified as a complex one since it has been discovered that its genome encodes not only structural proteins, but also at least three regulatory and accessory proteins. Between two long terminal repeats (LTRs) located at 5ʹ and 3ʹ like for all retroviruses, there are 4 ORFs encoding gag proteins, protease (PR), pol proteins and env proteins. MMTV LTRs encode two additional genes, *sag* and *rem*, which are translated in a superantigen and an RNA exporting protein, respectively. Sag is a transmembrane protein with an essential role in tumor development, since it is responsible of the efficient transmission of viral particles from the gut to the mammary gland: its action is essential to the lymphocytes amplification of cognate cells deriving from the Sag-MHC complex recognition by specific T-cells, but it is not required for the initial infection [316]. Rem, which should be associated with a Rem-responsive element located on MMTV RNA, is responsible for the transport of unspliced viral RNAs [317]. Furthermore, MMTV LTRs are also important since they encode hormone response elements (HREs) upregulating viral production [318, 319], in addition to negative regulatory elements (NREs) inhibiting viral transcription [320], and a transcription enhancer factor-1 (TEF-1) binding site, involved in tumorigenesis [321].

Over time, an MMTV variant, the type B leukemogenic virus (TBLV), was discovered. This is involved in the development of lymphomas, particularly T-type ones, in mice [322, 323]. It is the result of defined LTR modifications, such as the loss of NREs, multiplication of regulatory elements, and the coding of T cell-specific transcriptional enhancers [324, 325]. These modifications cause the virus to shift its ability from causing mammary cancer to inducing lymphoma.

The viral cycle starts when exogenous virions in nursing mice milk reach the gut of suckling pups, infecting first dendritic cells and B lymphocytes in the Peyer’s patches. These cells process the Sag antigen and expose it on their surface, in the context of the major histocompatibility complex (MHC) class II. Sag-activated T-lymphocytes, in turn, further stimulate the proliferation of dendritic cells, B- and T-lymphocytes, and establish a reservoir of both permissive and infected cells. Hence, the virus exploits immune cells to reach its target, the mammary gland, where infection of mammary epithelial cells occurs during hormonal stimulation typical of puberty and pregnancy, when they are prone to divide [316]. For this reason, there is a period of latency between the time of ingestion of infected milk and tumor development. Once hormonal stimulation starts, MMTV binds to host cellular Transferrin Receptor 1 (TfR1) and passes the cell membrane within a low-pH endosome. Inside the cell, the viral genome is reverse-transcribed and delivered to the nucleus, where integration into the host genome as proviral DNA occurs [316]. To produce virions, viral RNA has to be translated, and both translation products and progeny RNAs are assembled into virions and released through budding from the host cells. On the other hand, amplification of the virus is necessary for tumors to develop. It is believed that MMTV infects multipotent mammary stem cells during hormonal circumstances, increasing the regenerative activity of the mammary gland [326, 327]. At first, MMTV causes a hormone-dependent hyperplastic alveolar nodule (HAN), followed by a hormone-independent phase of tumorigenesis [316, 328]. It has been suggested that MMTV-induced mammary carcinoma is a monoclonal tumor, with all neoplastic cells originating from one individually transformed and expanded stem cell [329–334]. MMTV is classified as a non-acutely transforming retrovirus, since it does not contain oncogenes. To develop cancer, the virus has to integrate its genome close to cellular proto-oncogenes, which are activated and overexpressed through the action of LTR promoters or enhancers. Particularly, the most common integration sites (CIS) involved in tumorigenesis of MMTV-related mammary tumors are members *of wnt, fgf, rspo, notch4/int3,* and *eIF3e/int6* [316, 334–336]. Activation of multiple genes is required for tumorigenesis [316], leading to disruption of cell signaling pathways, dysregulation in cell proliferation and differentiation and, finally, to tumor development. TBLV, the MMTV variant causing lymphoma, has some structural differences in U3 region of LTRs allowing a different target. Particularly, TBLV U3 region is characterized by a deletion of NREs and multiple flanking sequences encoding for lymphocyte-specific transcriptional enhancers [324]. These structural modifications are per se sufficient to cause lymphoma, rather than mammary carcinoma [325], although the mechanisms of viral oncogenesis remain the same [337–340]. TBLV CIS are members of *myc, rorc, notch1* and *tblvi1* gene families. Furthermore, it has been suggested that, differently from mammary tumors, MMTV lymphomas are polyclonal neoplasms and require additional infection and proviral integration events for their development [325].

Although a considerable number of studies have been conducted, evidence of the hypothetical involvement of an MMTV-like virus in neoplasms of human and domestic animals is still weak, as reported by Amarante et al., Szabo et al., and Parisi et al. [341–343]. Specifically, the suggestion of such etiology in the development of BC has been the subject of a long controversy between researchers who support this hypothesis and those who do not [341]. However, over time, compelling evidence has accumulated both in vivo and in vitro, and it is now generally accepted that an MMTV-like virus, known as Human Mammary Tumor Virus (HMTV), is associated with BC. The mechanisms of oncogenesis and common viral integration sites have been studied [343].

It has been highlighted that MMTV infects a variety of murine organs, such as kidney, salivary glands, and the male genital system [344–346], and that TBLV is involved in T-cell lymphoma development [322, 323, 347, 348]. Similarly, in humans, sequences of a virus very closely resembling MMTV have been found in other tissues, in addition to T cells, which has given rise to the hypothesis that an MMTV-like virus may be also associated with human lymphomas [349–351], hepatic carcinomas [352], and other diseases affecting the liver, such as primary biliary cirrhosis [353–355]. MMTV-like virus has been associated not only with T-cell lymphomas, as in mice [356–358], but also with B-type lymphoma. Moreover, MMTV-like sequences have been found in other neoplasms, such as those in the ovary, prostate, skin [359], and endometrial carcinoma [360], although the role of the virus in the development of these human cancers still needs to be clarified. Viral sequences have also been found in saliva, blood, and milk. It has been suggested that the presence of the virus in saliva may follow inter-human spread [361].

In an effort to provide further insight into the epidemiology of this virus, Stewart et al. [362, 363] noted that North West Europe, the European area with the highest incidence of BC, matched geographically with areas where a particular mouse strain, *Mus domesticus*, was a resident species. This evidence led to the suspicion that the virus could spread from mice to humans using domestic animals, such as cats and dogs, as intermediary hosts. In recent years, veterinary medicine studies focused on the search for a putative MMTV-like virus in dogs and cats. The first evidence of MMTV-like sequences was found in 2005, when MMTV-like sequences were amplified from the thymus of a kitten and the spleen of an adult cat [364]. In 2012, Hsu et al. investigated mammary tumors from 145 dogs and 11 cats, targeting MMTV-like *env* and LTR sequences. They found a prevalence of 3.49% (3/145) and 18.6% (16/145) for *env* and LTR sequences in dogs, respectively, and 22.22% (2/11) for both targets in cats [365]. The amplificon sequences shared 94% and 98% similarity with MMTV and HMTV, respectively. Additionally, they detected the presence of these sequences in normal mammary tissue from both cats and dogs. Two subsequent studies confirmed the presence of an MMTV-like sequence sharing high homology with MMTV and HMTV in feline mammary tumors, with a prevalence of 7% (7/86) [366] and 12.5% (3/24) [367], but not in healthy tissue from both cats and dogs, nor in canine mammary tumors. Preliminary research on feline lymphomas showed that MMTV-like sequences may also be found also in this kind of neoplasm [368]. Particularly, the authors found a 9.4% prevalence (5/53) of MMTV-like sequences and a strong association with lymphoma localized in the nasal cavity, since two out of the three nasal lymphomas included in the study were found to be positive. However, all these are only preliminary results on an interesting topic that requires further investigation. Due to the evidence of MMTV involvement in carcinogenesis in humans and mice, any similar etiological agents should be identified in domestic animals, to develop coordinated therapies and prevention measures and to investigate their hypothetical zoonotic potential.

## Conclusions

Viral oncology is an emergent topic continually under research. Knowledge about viruses involved in the tumor formation process in dogs and cats is essential, both as pathogens for their original host and as animal models for human diseases. Regarding the last aspect, particular attention should be paid to PV-positive FOSCCs, due to the most recent evidence suggesting that the cat may be a potential animal model for PV-induced HNSCCs in humans. However, knowledge on oncogenic viruses of companion animals relies almost entirely, although with some exceptions, on individual studies and research carried out by isolated working groups, lacking in some cases an overall picture. Moreover, in this field, a joint effort of veterinary and human medicine is crucial since the results obtained by investigating the molecular mechanisms may often be of interest to both species. With this work we would like to give an overview of the progress made in canine and feline oncovirus field, also highlighting the most relevant aspects of their human counterpart. Further studies on the pathophysiological mechanisms underlying the interactions between viruses and cancer are encouraged. They would support the hypothesis of a role of putative oncoviruses in tumor formation, solidify knowledge about already proven oncogenic viruses, and develop new medical treatments in human and veterinary medicine.

## Data Availability

Not applicable.
